# Simple decompression vs. subcutaneous anterior transposition of the ulnar nerve: the 2025 update on the optimal treatment for cubital tunnel syndrome

**DOI:** 10.1016/j.xrrt.2025.100630

**Published:** 2025-11-29

**Authors:** Bishnu Pokharel, Chiara Fossati, Sailesh Bhattarai, Faizan Vaja, Andrew Kemetse, Aurelien Traverso, Pietro S. Randelli

**Affiliations:** a U.O.C. 1^a^ Clinica Oropedica, ASST Centro Specialistico Ortopedico Traumatologico Gaetano Pini-CTO, Milan, Italy; b B. P. Koirala Institute of Health Sciences, Dharan, Nepal; c Laboratory of Applied Biomechanics, Department of Biomedical Sciences for Health, Università degli Studi di Milano, Milan, Italy; d Department of Orthopaedics and Traumatology, Centre Hospitalier Universitaire Vaudois (CHUV), Lausanne, Switzerland; e Research Center for Adult and Pediatric Rheumatic Diseases (RECAP-RD), Department of Biomedical Sciences for Health. Università degli Studi di Milano, Milan, Italy

**Keywords:** Ulnar nerve, Cubital tunnel syndrome (CuTS), Simple decompression (SD), Subcutaneous anterior transposition (SAT), Recurrence, Revision surgery

## Abstract

This 2024 update aims to compare the clinical outcomes, recurrence, and revision rates between simple decompression (SD) and subcutaneous anterior transposition (SAT) of the ulnar nerve for treating cubital tunnel syndrome (CuTS). A systematic review of articles from PubMed, Scopus, and Google Scholar was performed in accordance with Preferred Reporting Items for Systematic Reviews and Meta-Analyses guidelines. We selected all English-language comparative studies published from January 2000 to June 2024, comparing SD and SAT concerning postoperative outcomes of surgical treatment for CuTS. Ten articles met the inclusion criteria. We found no statistical difference in the clinical outcomes of SD and SAT. Two studies indicated SD was superior to SAT. Four studies concluded that both techniques were equally effective for CuTS treatment. Three studies were inconclusive, and one study reported *in situ* decompression as inferior to SAT, with a high recurrence rate. As of 2025, the surgical approach to CuTS remains consistent with current practice. Both SD and SAT of the ulnar nerve continue to demonstrate good clinical outcomes. Although SD may be associated with higher rates of recurrences and revision surgeries, no clear superiority of one technique over the other has been established.

Cubital tunnel syndrome (CuTS) is the second most common entrapment syndrome after carpal tunnel syndrome.^7^ Generally, surgery is indicated when nonoperative conventional treatment fails. Three surgical techniques, with some variations, are commonly used to treat CuTS. They are: (1) *in situ* decompression, (2) anterior transposition, and (3) medial epicondylectomy.^8^

Simple decompression (SD) and subcutaneous anterior transposition (SAT) of the ulnar nerve are the most widely performed surgery for the treatment of CuTS. SD is gaining popularity because of its high cosmetic value, less surgical time, and early return to work. However, it is not without complications. Because of small incisions, there will be inadequate decompression and persistence of symptoms even after surgery. Similarly, they can cause postsurgical scars in decompressed sites and recurrence of symptoms after surgery.^5^

Abourisha E. et al^2^ in their systematic review and meta-analysis of 10 randomized controlled trials to discern superiority between open in situ, endoscopic, and anterior transposition (subcutaneous or submuscular techniques) concerning the primary outcome of response to treatment and secondary outcomes, which include complications, postoperative chronic pain visual analog scale, return to work, and reoperation. They did not find any differences between techniques concerning return-to-work rates or reoperation rates. Heterogeneity in the outcome measures and the need for better research was pointed out. But they concluded that endoscopic decompression was found more hazardous than open *in situ* decompression with medial epicondylectomy.

At the same time, SAT has been used to treat CuTS for many years since it was first successfully performed and described by BF Curtis in 1898. Although it has a low recurrence rate and better functional outcomes, big surgical scars and delayed return to work are major issues.^6^

Wade RG et al^18^ in their systematic review and meta-analysis of 30 studies aimed to evaluate which operation for CuTS is associated with the greatest likelihood of symptomatic cure. They compared *in situ* decompression (open, minimally invasive, or endoscopic), with or without medial epicondylectomy, and an anterior subcutaneous, subfascial, intramuscular, or submuscular transposition. They found that open *in situ* decompression (with or without medial epicondylectomy) appeared to be the safest operation and also was associated with the best outcomes for patients with primary CuTS.

In these high-quality meta-analyses, they have pointed out the serious need to define the condition, standardized the operational definition of the procedures, and outcome measures. There is a lack of guidelines for selecting appropriate techniques for the treatment of CuTS. At present, the choice of the surgical technique is based on the surgeon's preference, their expertise, and national guidelines in different countries. This study aimed to perform a systematic review of the literature, comparing the clinical outcomes, recurrence, and revision rates of SD and SAT for treatment of CuTS.

## Material and methods

### Literature and database search

Original comparative studies of level I-IV on the surgical treatment of CuTS by SD and SAT of ulnar nerve, whether prospective or retrospective published between January 2000 and June 2024, were included. Case reports, articles that are case series with <15 patients; SD with medial epicondylectomy, systematic reviews and meta-analysis abstracts without full text, and clinical studies with a minimum average follow-up of <6 months were excluded.

A systematic review of scientific articles listed in medical databases (PubMed, Scopus, and Google Scholar) was performed in September 2024, according to Preferred Reporting Items for Systematic Review and Meta-Analyses (PRISMA) guidelines. The search consisted of Medical Subject Heading words: (cubital tunnel syndrome) AND (anterior transposition) AND/OR (simple decompression/*in situ* decompression) OR recurrence. The search was restricted to English language literature. The search strategy was guided by the population, intervention, comparison, outcomes, study design principles that focused on the population with CuTS, with interventions of either SD or anterior transposition of ulnar nerve in the hospital settings.

### Selection process data extraction

**Figure 1:**
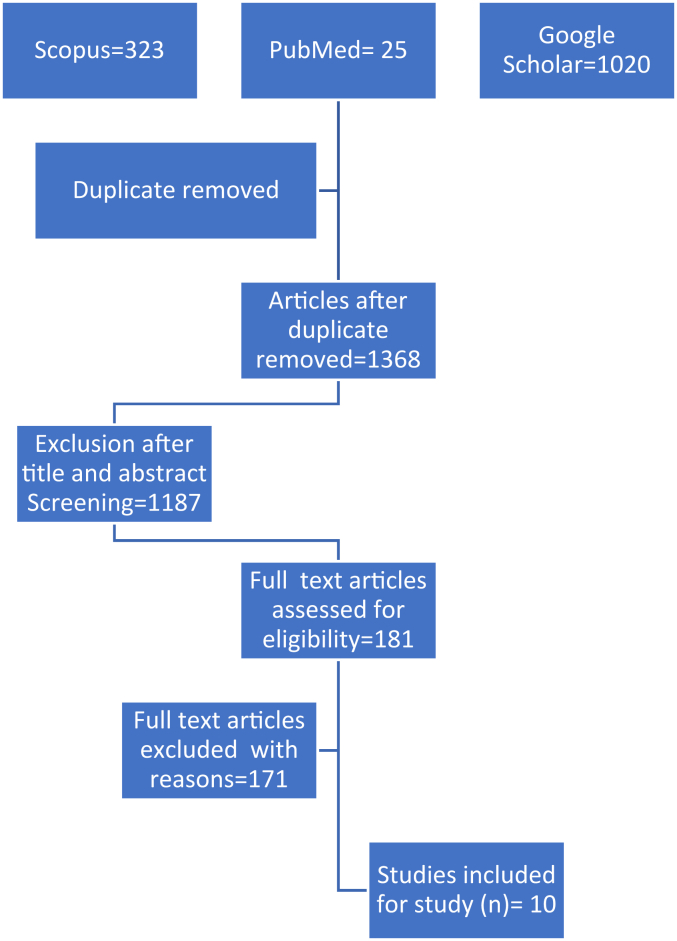
PRISMA flow chart of selection of the studies. *PRISMA*, Preferred Reporting Items for Systematic Review and Meta-Analyses.

Titles and abstracts were screened for relevance by all the authors. If necessary, the complete article was reviewed to reach a decision. We also reviewed the references of papers to locate additional studies. PRISMA flowchart for the process of selection of article is shown in Figure 1. All the articles retrieved from the keyword search were entered in Microsoft Excel. Three of the authors independently reviewed the titles and abstracts of the retrieved publications in the first step to exclude irrelevant studies as per the inclusion and exclusion criteria. In the second step, each publication was read in full text by 2 of the 7 reviewers in our team. In the next step, the eligibility assessment and data extraction of data from qualified articles were performed by 2 independent reviewers. Quantitative and qualitative data regarding CuTS were extracted. Disagreements during the process were resolved by discussion between the reviewers.

Our initial search of the 3 databases yielded a total of 1,368 papers after duplicates were removed. Full-text articles assessed for eligibility were 181; after screening, 10 studies met the inclusion criteria. All the comparative studies on the outcome of CuTS following surgical treatment by *in situ* decompression and anterior transposition published between Jan 2000 and June 2024 in the English language, whether prospective or retrospective, were included.

The flowchart in Figure 1 displays the results of each step of the screening process with the number of studies and reasons for each decision made. Our results are reported by the PRISMA reporting guidelines.

### Risk of bias assessment

The risks of methodological bias was assessed by 2 out of the 7 authors independently using the risk of bias in nonrandomized studies of interventions (ROBINS-I) assessment tool for the retrospective studies. The ROBINS-I has 7 domains to assess the risk in observational studies: bias due to confounding effect, bias in selection of participants, bias in classification of interventions, bias due to deviations from intended interventions, bias due to missing data, and bias in selection of reported result. Each article was thoroughly read before answering the items in each domain where responses were recorded in “yes, probably yes, no, probably no, and no information.” The judgment on whether low risk of bias, moderate risk of bias, serious risk of bias, or critical risk of bias were reached with the help of “Reaching risk of bias judgments in ROBINS-I″ table for each domain of bias. The disagreements in judgment of bias were resolved by discussion among the 2 authors involved in methodological bias assessment. Similarly, risk of bias assessment for one prospective clinical trial was assessed by Cochrane risk of bias tool for randomized trials, which assesses the biases on 5 domains: bias due to randomization, bias due to intended interventions, bias due to missing data, bias due to measurement of outcomes, and bias due to selection of reported results.

## Results

This systematic review included retrospective comparative observational studies and an intervention study (Table I). These studies compared SD and SAT, with outcomes measured by clinical improvement, patient satisfaction, or other primary outcome measures in the treatment of CuTS.

**Table I:** Descriptive background and preoperative information of the study population in the studies involved.

Author/yr	Study design and level of evidence	No of patients in *in situ*/subcutaneous anterior transposition	Mean (standard deviation) age of the group (yr) SD/AT	Mean (standard deviation) duration of symptoms before surgery (mo)	Etiology	Preoperative assessment tool/classification system	Severe preoperative classification% *in situ*/SAT
Walid K. Abouzeid (2022)^1^	Retrospective cohort/III	38/41	40 (±10)/42 (±14)	19 (±8)/±23 (±9)∗	Idiopathic	Dellon	18.4/29.3
R. M. Lanzetti (2021)^10^	Retrospective cohort/III	41/66	54 (among total)	6 (among total patients)	Mostly idiopathic, injury, arthritis, valgus deformity, benign neoplasm (nonspecified)	McGowan	33/34
Duncan Van Nest (2020)^16^	Retrospective cohort/III	979/405	53.8 (±14.5)	NI	Idiopathic	McGowan	-
Douglas T Hutchinson (2019)^8^	Retrospective cohort/III	73/59	48/47 matched	NI	Idiopathic	McGowan	Similar across the interventions
Izadpanah Ali (2019)^9^	Retrospective cohort/III	17/47	54/54	NI	Nonspecified	McGowan	All stage III cases taken
Marco Suosa (2014)^15^	Retrospective cohort/III	64/33	51.2 (±14.7)/(53.3 ± 15.6)	NI	Idiopathic	Modified McGowan	29.7/33.3
G. Bacle (2014)^2^	Retrospective multicenter/III	48/229	48 (28-71)/54 (19-87)	94 (66-110)/50 (9-127)	Nonspecified	McGowan Goldberg	27/21
Grigorious I.Mitsionis (2010)^11^	Retrospective/III	31/37	51,751	15 (2-48) all cases	Idiopathic	Modified McGowan Goldberg score	12.9/40.54∗
Y Ramguthy (2009)^12^	Retrospective cohort/III	47/55	46 (19-78)/43 (20-68)	NI	Nonspecified	No severity check was done	NI
Ronalds Bartel (2005)^3^	Prospective clinical trial/I	75/77	47.2 (±12.9/47.1 (±))	8.6 ((±10.1)/8.9 (±10.4))	Idiopathic	Clinical	The average score was not significantly different

*SD*, simple decompression; *SAT*, subcutaneous anterior transposition; *NI*, not indicated.

∗ *P* value < .05.

Using the original search strategy, 181 articles were identified in the medical database, of which 10 met the inclusion criteria and were included in this systematic review. Nine of the 10 studies were retrospective, and only one was a prospective clinical trial. In the retrospective studies,^1^^,^^3^^,^^9^, ^10^, ^11^, ^12^, ^13^^,^^16^^,^^17^ the average follow-up period was 6 years (ranging from 22- 10 years), and in the prospective study,^4^ the average follow-up period was 3 years.

McGowan or modified McGowan was used as a preoperative severity assessment tool in 6 studies.^1^^,^^3^^,^^9^^,^^10^^,^^16^^,^^17^ Dellon classification was used in one study,^1^ and nerve conduction velocity/electromyography in another.^4^ Preoperative severity was not checked in one study.^13^ One study^17^ assessed only revision surgery as the main outcome. For postoperative outcome assessment, the Bishop classification score was used by 2 studies,^1^^,^^13^ Wilson and Kraut by 3;^11^^,^^12^^,^^16^^,^ McGowan by one;^3^ Clinical Telephone by one;^10^ and clinical outcome assessment by 2 studies.^4^^,^^10^ One study^10^ exclusively selected patients with CuTS of McGowan III. In all other studies, patients were at different stages of the disease, but these differences were statistically insignificant. Six studies clearly reported idiopathic CuTS,^1^^,^^4^^,^^10^^,^^12^^,^^16^^,^^17^ while 4 studies mentioned CuTS with or without obvious pathology.^3^^,^^8^^,^^10^^,^^13^ In all studies, patient selection for SAT or SD was at the surgeon's discretion.

There was no uniformity in the surgical technique, especially for *in situ* decompression, and no standard case definition for SD. The length of the incision and the level and extent of decompression varied across studies for *in situ* decompression, except for 3 studies,^6^^,^^12^^,^^13^ where these parameters were similar. In 2 studies,^3^^,^^9^ the incision size was smaller for *in situ* decompression. Five studies did not mention surgical details. The decision to perform anterior transposition of the ulnar nerve was made intraoperatively in 3 studies because of instability^12^^,^^13^ or because the nerve bed was not suitable^11^^,^^13^ to leave the nerve after decompression, in fear of adhesion, so they decided anterior transposition of ulnar nerve.

### Risk of bias assessment

This study considered preoperative severity and comorbidity as important confounders, along with symptom duration and case epidemiology. These were also considered confounders requiring adjustment for decision-making. Six studies, including the clinical trial, had a severe risk of bias,^1^^,^^3^^,^^4^^,^^11^, ^12^, ^13^ whereas 4^9^^,^^10^^,^^16^^,^^17^ were assessed as having a moderate overall risk. By the nature of their design, most observational studies were susceptible to confounding bias. This was because some studies lacked information about major confounders, and some did not adjust for them, even when the confounding variables were significantly different between groups (Table II, Table III).

**Table II:** Risk of bias assessment for observational studies.

Risk of bias	Confounding	Selection of participants	Classification of interventions	Deviation from intended interventions	Missing data	Measurement of outcomes	Selection of reported result	Overall bias assessment
Walid K. Abouzei (2022)^5^	S	M	M	L	M	M	M	S
R. M. Lanzetti (2021)^6^	S	S	S	L	L	M	M	S
Duncan Van Nest (2020)^7^	L	M	M	L	L	M	M	M
Douglas T Hutchinson (2019)^8^	L	M	M	M	L	M	L	M
Izadpanah Ali (2019)^9^	M	M	M	M	M	M	L	M
Marco Suosa (2014)^10^	M	M	M	L	NI	M	L	M
G. Bacle (2014)^11^	M	M	M	L	S	M	M	S
Grigorious I.Mitsionis (2010)^12^	S	M	M	M	L	M	M	S
Y Ramguthy (2009)^13^	S	S	M	M	L	M	L	S

*S*, severe risk of bias; *M*, medium risk of bias; *L*, low risk of bias; *C*, critical risk of bias.

**Table III:** Risk of bias assessment for randomized controlled trial.

Risk of bias	Bias due to randomization process	Deviation from intended intervention	Missing outcome	Bias in measurement of outcomes	Selection of reported result	Overall bias
Ronalds Bartel (2005)^14^	L	L	SC	L	L	SC

*S*, severe risk of bias; *SC*, some concern; *L*, low risk of bias; *C*, critical risk of bias.

For example, the study by Walid K Abouzie et al^1^ revealed similar preoperative backgrounds, except for significantly greater CuTS severity in the SAT group and a longer mean duration of symptoms before surgery. However, these preoperative confounders were not adjusted in the analysis. The study by R. M. Lanzetti et al^11^ included cases with various etiologies, such as injury, arthritis, valgus deformity, and benign neoplasm, but there was no information regarding the distribution of etiology between the groups. Intervention selection was based on indication. There was also no information regarding the distribution of the mean duration of symptoms before surgery or the distribution of comorbidities between the groups. The study by Duncan Van Nest et al^17^ had no information regarding preoperative disease severity, symptom duration, etiology, or comorbidity.

The major preoperative variables and other confounders were mentioned and matched in the study by Douglas T. Hutchinson et al^9^ However, selection of participants, adjustment of missing data, measurement of outcomes, and selection of reported data were moderately biased. The study by Izadpanah Ali^10^ also had most preoperative confounders matched, but other biases were moderate due to a lack of information regarding the major outcome. Similarly, the study by Marco Suosa et al^16^ had a similar distribution of preoperative disease severity, age, and etiology among the groups; however, the study was moderately biased in confounding, selection of participants, and classification of intervention. The study by G. Bacle et al^3^ was moderately biased on confounding adjustment, selection of participants, and classification of interventions but was seriously biased in addressing missing data. Studies by Y. Ramguthy B. et al,^13^ I. Mitsionis et al,^12^ and R. M. Lanzetti et al^6^ made an intraoperative decision to perform anterior transposition as indicated. Preoperative severity was significantly higher among SAT cases but was not adjusted in the final analysis. The study by Y. Ramguthy B et al^13^ was seriously biased on confounding adjustment and selection of participants. The final randomized clinical trial had some concerns over missing data, as it was not appropriately addressed.

However, in most observational studies, the start of follow-up coincided with the intervention. Similarly, most studies properly defined the intervention, and no deviation from the intended intervention was observed. Furthermore, most studies reported the primary findings, and by nature, the interventions were open, which could have had some minimal impact on the final outcome (Table IV).

**Table IV:** Intraoperative and postoperative findings of the surgical procedures in the studies involved.

Authors	Mean (standard deviation)/median (IQR) follow-up (mo) *in situ*/SAT	Preoperative Comorbidities	Recurrence/revision surgery (%) *In situ*/SAT	Hospital stay *in situ*/SAT (%)	Postoperative assessment tool	Excellent and good outcome (%) *in situ*/SAT	Postoperative complications
Walid K. Abouzeid (2022)^1^	28	Not reported	15.8/2.4∗	1.5 ± 0.2/3 ± 0.6∗	Modified bishop rating	94.7/97.5	21/26.8
R. M. Lanzetti (2021)^10^	3/3	24.3% in total diabetes, arthritis, autoimmune disease	6 (overall)	NI	Wilson and Krout	71/74	13 (overall)
Duncan Van Nest (2020)^16^	52	NI	3.1/2.2	2.2	Revision surgery	NI	-
Douglas T Hutchinson (2019)^8^	117 (53)/90/(47)	comorbidities matched	25/12∗	12	Reoperation	-	-
Izadpanah Ali (2019)^9^	32.4 ± 16	Comorbidity reviewed but distribution not mentioned	21/15	23	Clinical	-	Not compared
Marco Sousa (2014)^15^	9.3 (4-29)/11.7 (6-33)	NI			Modified Wilson and Krout	67.2/69.7	
G. Bacle (2014)^2^	94 (66-110)/50 (9-127)	NI	2.5/0	0	McGowan Goldberg	85.4/73.80	0/1.3
Grigorious I.Mitsionis (2010)^11^	37 (24-120) all cases	Hypothyroidism, diabetes, and chronic renal failure excluded	6.4/2.7	2.7	Modified Wilson and Krout	84/62	Grip key pinch strength were comparable
Y Ramguthy (2009)^12^	3.6 (±2)/4.3 (±1)† time to recover	NI	NI	NI	Bishops	94/93	4.25/3.51
Ronalds Bartel (2005)^3^	12/12	Comorbidities and symptoms excluded	4/7.8	7.8	Clinical	65.33/70.13	9.6/31.1∗

*SAT*, subcutaneous anterior transposition; *IQR*, interquartile range; *NI*, not indicated.

∗ *P* value < .05, *P* value <.001∗∗.

† Mean time to recover.

## Discussion

In this systematic review, we evaluated and compared the clinical outcome, recurrences, and reoperations rate between *in situ* decompression and subcutaneous ulnar nerve transposition (UNT) in CuTS.

The 2 surgical procedures were shown to be safe and effective. Moreover, no statistical differences were found between the 2 techniques in term of clinical outcomes. However, the authors in the 4 studies preferred to perform *in situ* decompression because of its technical simplicity and low local wound complications.^1^^,^^4^^,^^12^^,^^13^ The 2 studies demonstrated that SD was associated with improved functional outcomes and lower complication rates compared to SAT.^4^^,^^13^ The 4 studies concluded that both techniques were equally effective for the treatment of CuTS.^1^^,^^3^^,^^12^^,^^16^ The 3 studies were not able to draw any conclusion,^10^^,^^11^^,^^17^ and in one study author showed *in situ* decompression to be inferior to the SAT of ulnar nerve with high recurrence rate.^9^

Majority of the studies have not mentioned the patient selection criteria and allocation to surgery. There was no standard case definition of *in situ* decompression. Some surgeons made long incisions just like anterior transposition where recurrence and revision are similar.^11^, ^12^, ^13^ Some surgeons preferred small incisions, where recurrence and revision are high.^3^^,^^9^ It has was clearly evident from these studies if the site of compression could be localized prior to surgery, decompression with a small incision would be adequate, but if the site of compression were uncertain, then large incision with decompression from the Struthers fascia to Osborn fascia would be better. If the chances of scarring were high following decompression, ulnar nerve was unstable, or there were other pathologies that increases the chances of recurrence, then SAT would be better. The difference in the outcome of surgical procedures in the treatment of CuTS, especially SD and anterior transposition is not significant as reported by the past studies. So, generalization is not possible because of inconsistencies in defining the procedure and variations in the surgical procedures in different. Interpretation of data is further limited by inconsistencies in reporting across individual studies. There is variability in many aspects of study design including patient selection criteria, sample size, length of follow-up, and outcome measures utilized.

In the pool of systematic review on SD vs. SAT of ulnar nerve for the treatment of CuTS in this study, we found that SD cannot be the substitute for SAT of ulnar nerve for the treatment of CuTS, which other study failed to mention.

In a meta-analysis of 4 randomized controlled trials comparing SD with anterior UNT (2 submuscular and 2 subcutaneous) conducted by Micheal Zlowodzki et al,^19^ found that there is no difference in motor nerve conduction velocities or clinical outcome scores between SD and UNT for the treatment of ulnar nerve compression at the elbow in patients with no prior traumatic injuries or surgical procedures involving the affected elbow. They suggested that SD of the ulnar nerve is a reasonable alternative to anterior transposition for the surgical management of ulnar nerve compression at the elbow.

Joseph Said et al,^15^ in their meta-analysis of 17 studies, found no statistically significant difference in clinical improvement and revision surgery with SD and anterior transposition of the ulnar nerve. However, there were significantly more complications with ulnar nerve transpostion. But they have also mentioned that the current body of evidence regarding CuTS lacks prospective, randomized, controlled trials, uniform reporting of indications, and standardized outcome scoring.

Adam Carlton et al^6^ conducted systematic review to provide an updated summary of the current literature on outcomes for various surgical treatments for CuTS. They reviewed articles published on *in situ* decompression (minimal incision, open, and endoscopic *in situ* decompression) and anterior transposition of ulnar nerve (subcutaneous, intramuscular, and submuscular). In their study they found varying but comparable levels of success among all surgical techniques reviewed, but quantitative comparisons were difficult because different scoring scales were utilized for measuring severity of disease and outcome of surgeries. They concluded that none of the techniques demonstrated universal superiority above all others, but all appear to be effective in the treatment of CuTS. The only consensus seems to be that transposition is preferred where the ulnar nerve tends to subluxate either on preoperative or intraoperative examination.

Ruettermann M. et al^14^ in his systematic review, “Challenging the dogma: anterior transposition of the ulnar nerve is indicated in recurrent cubital tunnel syndrome,” wrote “a meta-analysis was not possible due to selection bias and disparity of outcome measurements of the studies. However, no robust evidence that supports the need of an anterior transposition of the ulnar nerve in recurrent cubital tunnel syndrome over an *in situ* decompression was found.”

This analysis has several important limitations. The included studies showed considerable variability and potential bias, limiting the generalizability of the findings. No strict criteria regarding journal type (eg, peer-reviewed, impact factor) were applied, and reporting of functional outcomes and complication rates was inconsistent, further restricting the precision of conclusions.

## Conclusion

As of 2025, the surgical approach to CuTS remains consistent with current practice. Both SD and SAT of the ulnar nerve continue to demonstrate good clinical outcomes. Although SD may be associated with a higher rate of recurrences and revision surgeries, no clear superiority of one technique over the other has been established.
